# A One Health Approach to Investigating Cache Valley Virus, Arkansas, USA, July 2023^1^

**DOI:** 10.3201/eid3106.250052

**Published:** 2025-06

**Authors:** Ann Carpenter, Noah Kojima, Theresa M. Dulski, Amanda E. Calvert, Kristen L. Burkhalter, Jennifer R. Ballard, Keith Blount, Anna Fagre, Elisa Thrasher, Katelyn Lazenby, Stephen Hedges, Audrey Long, Kerri Miazgowicz, Erin Wood, Phillip Owens, C. Roxanne Connelly, Carolyn V. Gould, J. Erin Staples, Laura Rothfeldt, Stacey W. Martin, Joan Burke

**Affiliations:** Centers for Disease Control and Prevention, Atlanta, Georgia, USA (A. Carpenter, N. Kojima, T.M. Dulski, A. Fagre); Arkansas Department of Health, Little Rock, Arkansas, USA (T.M. Dulski, K. Blount, K. Lazenby, S. Hedges, L. Rothfeldt); Centers for Disease Control and Prevention, Fort Collins, Colorado, USA (A.E. Calvert, K.L. Burkhalter, A. Fagre, E. Thrasher, A. Long, K. Miazgowicz, C.R. Connelly, C.V. Gould, J.E. Staples, S.W. Martin); Arkansas Game and Fish Commission, Little Rock (J.R. Ballard); US Department of Agriculture Agricultural Research Service, Booneville, Arkansas, USA (E. Wood, P. Owens, J. Burke)

**Keywords:** Cache Valley virus, viruses, vector-borne infections, zoonoses, mosquitoes, arboviruses, animal health, Bunyamwera serogroup, sheep diseases, One Health, Arkansas, United States

## Abstract

Cache Valley virus (CVV), a mosquitoborne virus, can cause neuroinvasive disease in humans and adverse reproductive outcomes in sheep and goats. In 2023, CVV RNA was detected in an aborted lamb from a flock in Arkansas, USA. We conducted a One Health investigation to explore the potential effects of CVV in Arkansas.

Cache Valley virus (CVV), a mosquitoborne virus in the family *Peribunyaviridae*, genus *Orthobunyavirus*, and serogroup Bunyamwera, circulates among several vertebrate species, including sheep, deer, horses, and cattle (*1*–*3*). CVV has been isolated from multiple mosquito genera, but the primary vector species likely varies by geographic location (*1*–*6*).

Humans are susceptible to CVV and can develop asymptomatic infection or clinical disease ranging from febrile illness to meningitis and encephalitis. Although most CVV infections in animals are subclinical, infection in sheep and goats during pregnancy can cause abortions, malformed fetuses, and congenital abnormalities (*1*). Fetal deformities involve musculoskeletal and central nervous system malformations such as scoliosis, hydrocephalus, and arthrogryposis (*7*). Adverse fetal impact depends on the timing of infection during gestation (*8*).

The geographic distribution and burden of CVV disease among humans and animals is not well characterized (*2*,*4*,*9*). Since the initial identification of CVV in mosquitoes in Utah, USA, in 1956, the virus has been detected across North America, including the United States, Canada, and Mexico (*1*,*3*). Eight human disease cases have been reported in the United States, with likely exposures in North Carolina, Missouri, Wisconsin, Michigan, and New York (*10*–*12*); an additional case was associated with blood transfusion from an asymptomatic infected blood donor in Illinois (*13*). Seroprevalence studies have demonstrated widespread exposure to CVV in humans and animals (*2*,*3*).

In February 2023, CVV RNA was detected in tissues from an aborted lamb at a farm in central Arkansas, USA (farm A). The farmer reported an abortion storm with an attack rate of ≈30%. Previously, there had been 1 other report of CVV in Arkansas in 2020, a seropositive sheep from a flock in northwest Arkansas (farm B), located ≈60 miles northwest of farm A (Figure). The identification of an abortion storm in a flock potentially related to CVV raised concern about the potential risk for CVV to human and animal health. We investigated the occurrence and health risk for CVV among humans and animals in Arkansas.

**Figure:**
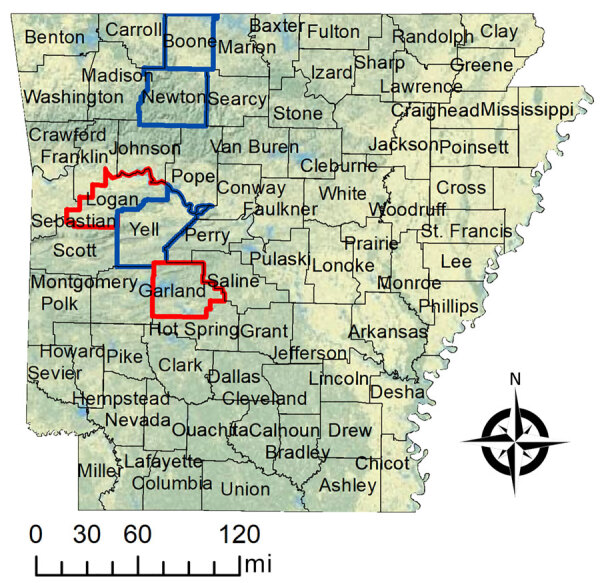
County locations for 2 farms with evidence of Cache Valley virus in sheep (red outline) and counties with specimens available from culled white-tailed deer (outlined in blue) for study of Cache Valley virus, Arkansas, USA, 2023.

## The Study

To investigate the effect of CVV on livestock, we acquired archived serum samples collected at breeding in August 2022 and 2 months’ gestation from sheep bred at farm B. Most of the flock were lambing for the first time, and the others did not have a history of fertility issues. Husbandry records included reproductive outcomes. To confirm the presence of CVV-specific antibodies in wild ungulates, we obtained archived serum samples from white-tailed deer culled in 2017 and 2020 during herd health assessments conducted in 3 counties in Arkansas (Figure).

We performed plaque reduction neutralization tests on sheep and deer serum diluted 2-fold from 1:10 to 1:1,280. We calculated maximal effective concentrations in which the serum dilution resulted in a 50% reduction in viral plaques (EC_50_) using virus neutralization curves with a 4-parameter logistic model. We defined seropositivity as EC_50_ >20. We also screened deer serum for neutralizing antibodies against Potosi and Jamestown Canyon viruses, 2 related orthobunyaviruses known to infect white-tailed deer in North America, including Arkansas (*1*).

To identify potential CVV vectors in Arkansas, we conducted dusk-to-dawn mosquito trapping using CO_2_-baited CDC miniature light traps, with and without light over 9 trap nights around farms A and B during the 2023 arboviral season (epidemiologic weeks 25–46). We homogenized captured female mosquitoes in cell culture medium/bovine albumin and extracted RNA for real-time reverse transcription PCR (*14*).

Meningitis and encephalitis are reportable conditions in Arkansas. To identify patients with meningitis and encephalitis of unknown etiology among cases reported to the Arkansas Department of Health (ADH) during January 1, 2022–July 9, 2023, we reviewed case data and applied standardized case definitions. Inclusion criteria were acute onset of fever and cerebrospinal fluid pleocytosis, defined as leukocytes (leukocyte) >5 cells/mm^3^. Patients classified as having encephalitis had >1 of the following: altered mental status, neurologic deficits, abnormal neuroimaging, abnormal electroencephalogram, or seizure. Patients classified as having meningitis had none of the characteristics of encephalitis and reported headache, stiff neck, or photophobia. To avoid capturing infections acquired at birth or in the hospital, we excluded patients <2 months of age and those whose initial CSF specimen was obtained on or after the third day of admission.

The Centers for Disease Control and Prevention deemed our investigation to be public health surveillance. We obtained archived serum samples from ewes under material transfer agreements with US Department of Agriculture and samples from deer from the Arkansas Game and Fish Commission. All specimens were collected under Institutional Animal Care and Use Committee–approved protocols held by the respective agencies.

We identified 39 ewes bred in August 2022 and lambed in mid-January to early February of 2023 with paired specimens at farm B. Of the 39 ewes, 8 (21%) were seropositive for CVV at breeding and 31 (79%) were seronegative. Of the seronegative ewes, 3 (10%) seroconverted during pregnancy; of those, 2 (66%) had adverse reproductive outcomes. The first ewe gave birth to a stillborn lamb with angular limb deformities and CVV detected in kidney tissue by PCR performed at Texas A&M Veterinary Medical Diagnostic Laboratory (College Station, Texas, USA) and a seropositive liveborn lamb reported to have weak legs. The second ewe had a stillborn lamb and a healthy-appearing liveborn lamb; no additional testing was performed. Of the 36 ewes that did not seroconvert during pregnancy, including the 8 seropositive at breeding, 6 (17%) had no pregnancy detected. Of the 30 with confirmed pregnancies, 10 (33%) had adverse reproductive outcomes: 3 embryo losses, 3 abortions, 3 stillbirths, and 1 congenital deformity.

Of 13 specimens obtained from deer culled in Yell, Boone, and Newton Counties (Figure), 11 (85%) were seropositive for CVV, including 1 that was seropositive only for CVV and 10 that were also seropositive for Jamestown Canyon or Potosi viruses (Table 1). One deer was seropositive for only Potosi virus. None of the 204 mosquito pools, representing 641 mosquitoes, tested positive for CVV (Table 2).

**Table 1:** Information on orthobunyavirus infections in white-tailed deer culled in 3 counties included as part of investigation of Cache Valley virus, Arkansas, USA, 2023*

County	CVV only	POTV only	CVV and JCV	CVV, JCV, and POTV
Yell, n = 5†	1 (20)	1 (20)	0	2 (40)
Boone, n = 1	0	0	0	1 (100)
Newton, n = 7	0	0	1 (14)	6 (85)
All, n = 13	1 (8)	1 (15)	1 (15)	9 (30)

*Values are no. (%) specimens with EC_50_ neutralizing antibodies >20. CVV, Cache Valley virus; EC_50_, effective concentration in which serum dilution resulted in a 50% reduction in viral plaques; JCV, Jamestown Canyon virus; POTV, Potosi virus. †One deer in Yell County tested negative for antibodies against all 3 viruses.

**Table 2:** Aggregated count of female mosquitoes trapped and tested as part of investigation of Cache Valley virus, Arkansas, USA, 2023*

Species	No. collected
*Aedes albopictus*	32
*Ae. atlanticus*	5
*Ae. cinereus*	1
*Ae. dupreei*	10
*Ae. infirmatus*	2
*Ae. triseriatus*	22
*Ae. vexans*	264
*Aedex *spp.	10
*Anopheles crucians *complex	3
*An. perplexens*	2
*An. punctipennis*	77
*An. quadrimaculatus*	5
*An. *spp.	3
*Culex erraticus*	113
*Cx. nigripalpus*	5
*Cx. quinquefasciatus*	13
*Cx. salinarius*	13
*Cx. tarsalis*	14
*Cx. territans*	1
*Culex *spp.	9
*Culiseta inornata*	3
*Psorophora columbiae*	9
*Uranotaenia sapphirina*	17
Unknown	8
Total	641

*All mosquito pools tested were negative for Cache Valley virus by real-time reverse transcription PCR.

We excluded 83 (40%) of 206 human patients reported with suspected meningitis and encephalitis from our study. Of the 123 remaining, 82 (67%) had complete records available and met case definition. Of those 82 cases, 4 (5%) had an unknown etiology, and none had a record of arboviral testing.

## Conclusions

This One Health investigation identified the presence of CVV and its likely effect on livestock health in Arkansas (*15*). Testing data for arboviral infections, including CVV infections, among human patients with meningitis and encephalitis were limited, suggesting human cases may be underdiagnosed.

We documented seroconversion during gestation in sheep, including those with adverse reproductive outcomes. Seropositivity among culled white-tailed deer provided further evidence of CVV transmission. We also detected antibodies to 2 related viruses in deer. More work is needed to understand the circulation and implications of CVV and other related orthobunyaviruses in domesticated and wild ungulates.

Vector surveillance activities did not identify CVV in collected mosquitoes. However, more extensive and sustained surveillance efforts over time are likely needed to detect virus in mosquitoes. Identifying reservoirs and local vectors of CVV will clarify opportunities for control measures to help prevent human and animal cases.
